# Esterified carotenoids are synthesized in petals of carnation (*Dianthus caryophyllus*) and accumulate in differentiated chromoplasts

**DOI:** 10.1038/s41598-020-72078-4

**Published:** 2020-09-16

**Authors:** Luna Iijima, Sanae Kishimoto, Akemi Ohmiya, Masafumi Yagi, Emi Okamoto, Taira Miyahara, Takashi Tsujimoto, Yoshihiro Ozeki, Nahoko Uchiyama, Takashi Hakamatsuka, Takanobu Kouno, Emilio A. Cano, Motoki Shimizu, Masahiro Nishihara

**Affiliations:** 1 Tokyo University of Agriculture and Technology, 2-24-16 Naka-cho, Koganei, Tokyo, 184-8588 Japan; 2 Institute of Vegetable and Floriculture Science, National Agriculture and Food Research Organization (NARO), 2-1 Fujimoto, Tsukuba, Ibaraki 305-0852 Japan; 3 Chiba University, 1-33 Yayoi-cho, Inage-ku, Chiba, 263-8522 Japan; 4 National Institute of Health Sciences, 3-25-26 Tonomachi, Kawasaki-ku, Kawasaki, Kanagawa 210-9501 Japan; 5 Japan Agribio Company Limited, 110-5 Itayamachi, Naka-ku, Hamamatsu, Shizuoka 430-0928 Japan; 6 Barberet & Blanc S. A., Camino Viejo 205, 30890 Puerto Lumbreras, Murcia Spain; 7 Iwate Biotechnology Research Center, 22-174-4 Narita, Kitakami, Iwate 024-0003 Japan

**Keywords:** Plant breeding, Plant molecular biology, Secondary metabolism

## Abstract

Although yellow and orange petal colors are derived from carotenoids in many plant species, this has not yet been demonstrated for the order Caryophyllales, which includes carnations. Here, we identified a carnation cultivar with pale yellow flowers that accumulated carotenoids in petals. Additionally, some xanthophyll compounds were esterified, as is the case for yellow flowers in other plant species. Ultrastructural analysis showed that chromoplasts with numerous plastoglobules, in which flower-specific carotenoids accumulate, were present in the pale yellow petals. RNA-seq and RT-qPCR analyses indicated that the expression levels of genes for carotenoid biosynthesis and esterification in pale yellow and pink petals (that accumulate small amounts of carotenoids) were similar or lower than in green petals (that accumulate substantial amounts of carotenoids) and white petals (that accumulate extremely low levels of carotenoids). Pale yellow and pink petals had a considerably lower level of expression of genes for carotenoid degradation than white petals, suggesting that reduced degradation activity caused accumulation of carotenoids. Our results indicate that some carnation cultivars can synthesize and accumulate esterified carotenoids. By manipulating the rate of biosynthesis and esterification of carotenoids in these cultivars, it should be feasible to produce novel carnation cultivars with vivid yellow flowers.

## Introduction

Flower coloration is an important aspect of the quality of ornamental plants. The major pigments in plants that determine flower color are flavonoids (including anthocyanins), carotenoids, betalains, and chlorophylls. Red and yellow colors in most plant species are produced by flavonoids, anthocyanins, and carotenoids. In plants belonging to the order Caryophyllales, except for those in the caryophyllaceous and pomegranate families, betalains are synthesized and accumulated; the red and yellow colors are produced by betacyanins and betaxanthins, respectively^1,2^. Carnations (*Dianthus caryophyllus* L.) are included in this exception and produce flower colors based on flavonoids such as anthocyanins and chalcones^3–5^. Yellow flowers in plants of the order Caryophyllales are due to betaxanthins or flavonoids; carotenoids have not been reported to underlie yellow or orange flowers in this order^1,2,6^.

Carotenoids are fat soluble pigments and are present in a wide range of plant species and produce yellow and orange coloring. In higher plants, carotenoids are synthesized and accumulated through multiple catalytic steps in plastids^7,8^. These plastids are classified on their structure as proplastids, etioplasts, chloroplasts, chromoplasts, and amyloplasts^9^. In the green tissues of higher plants, carotenoids are present in chloroplasts and have important functions in photosynthesis and protection against photooxidative damage^10,11^. The carotenoids lutein, β-carotene, violaxanthin, and neoxanthin are involved in photosynthesis and accumulate in green tissues^12,13^. In non-photosynthetic tissues, such as flowers and fruits, carotenoid contents and composition greatly differ from photosynthetic tissues even within species and these carotenoid compounds are accumulated in chromoplasts. An increase in the number and size of mature chromoplasts results in the accumulation of large amounts of carotenoids to produce colorful petals that attract animals and insects as pollinators^9,14^.

It has been demonstrated that the content and composition of carotenoids in petals are determined not only by carotenoid biosynthesis activity, but also by degradation activity and xanthophyll esterification activity^15^. Most of the genes encoding enzymes involved in carotenoid biosynthesis in higher plants have been identified^7,8,16^. The carotenoid content in petals largely depends on the transcriptional level of these carotenoid biosynthesis genes. In marigolds (*Tagetes erecta*), the high accumulation of total carotenoids in deep yellow petals is the result of a relatively high level of expression of the *deoxyxylulose 5-phosphate synthase* (*DXS*) and *phytoene synthase* (*PSY*) genes compared to expression levels in pale yellow petals^17^. During petal development, the increase in the carotenoid content is correlated with the upregulation of carotenogenic gene expression^18–21^. A second key factor that influences carotenoid accumulation in petals is the level of carotenoid degradation activity. In chrysanthemum (*Chrysanthemum morifolium*), lily (*Lilium brownii*), crocus (*Crocus sativus*), the cultivated brassica species, and petunia (*Petunia* × *hybrida*), all of the carotenogenic genes are expressed during carotenoid synthesis; however, synthesized carotenoids are rapidly degraded by the high levels of the carotenoid cleavage dioxygenase 4 (CCD4) enzyme, leading to white petals^22–26^. A third factor is xanthophyll esterification. In many plant species, xanthophylls comprise the majority of carotenoids in petals; xanthophylls are carotenoids with oxygenated residues. Most xanthophylls in flower and fruit tissues are esterified by fatty acids due to activity of the enzyme xanthophyll esterase (XES) that uses acyl-CoAs as donors^17, 27^. In tomato (*Solanum lycopersicum*), loss of XES activity results in the drastic reduction of xanthophylls in petals, indicating that esterification is important for xanthophyll accumulation^27^. High levels of esterified xanthophylls are present in petals of marigold, tomato, and calibrachoa (*Calibrachoa* × *hybrida)* and produce bright yellow petal coloration^15,27,28^. Esterified xanthophylls associate with lipoproteins called fibrillins and are sequestered into lipid droplets in chromoplasts called plastoglobules^29^. This sequestration mechanism is presumed to stabilize the esterified carotenoids and enables a large accumulation of carotenoids in chromoplasts.

Carnation flowers show a range of colors: red colors are produced by anthocyanins; cream colors are produced by flavonols; and green colors are produced by chlorophylls^29–31^. Yellow carnation flowers are produced by chalcone 2′-*O*-glucoside but not carotenoids^2,32,33^. Orange coloration is generated by the mixture of yellow, produced by chalcone 2′-*O*-glucoside, and red, produced by anthocyanin^6^. The synthesis and accumulation of esterified carotenoids have not been reported for yellow petals in plants of the order Caryophyllales that includes carnations^34^. Green petals in carnations accumulate a similar carotenoid composition as the leaves, although the content in petals is only one-tenth or less of that in leaves^34^. In the petals of cyanic and acyanic cultivars, very low levels of carotenoids are detectable; expression of genes for enzymes involved in carotenoid synthesis, such as *PSY* and *lycopene ε-cyclase* (*LCYE*), is significantly lower in these cultivars than in cultivars with green petals^34^.

The mechanisms of white and cream color development in carnation flowers have been analyzed in various carnation cultivars. In a survey of the relationship between flower colors and flavonoid compositions of carnation petals, we found that the cultivar ‘Club’, which has pale yellow flowers, did not have detectable levels of chalcone derivatives. Yellow pigments in petals of this cultivar could not be extracted by methanol but could be extracted by acetone, indicating that these pigments might be fat-soluble pigments, similarly to carotenoids. Here, we analyzed the pigments contained in the petals of ‘Club’ and confirmed that the pale yellow petal color was derived from carotenoids. To determine the mechanism of carotenoid accumulation in ‘Club’ petals, we compared carotenoid compositions in four carnation cultivars and analyzed their patterns of expression of genes for enzymes involved in pigment metabolism.

## Results

### Carotenoid content and composition

**Figure 1:**
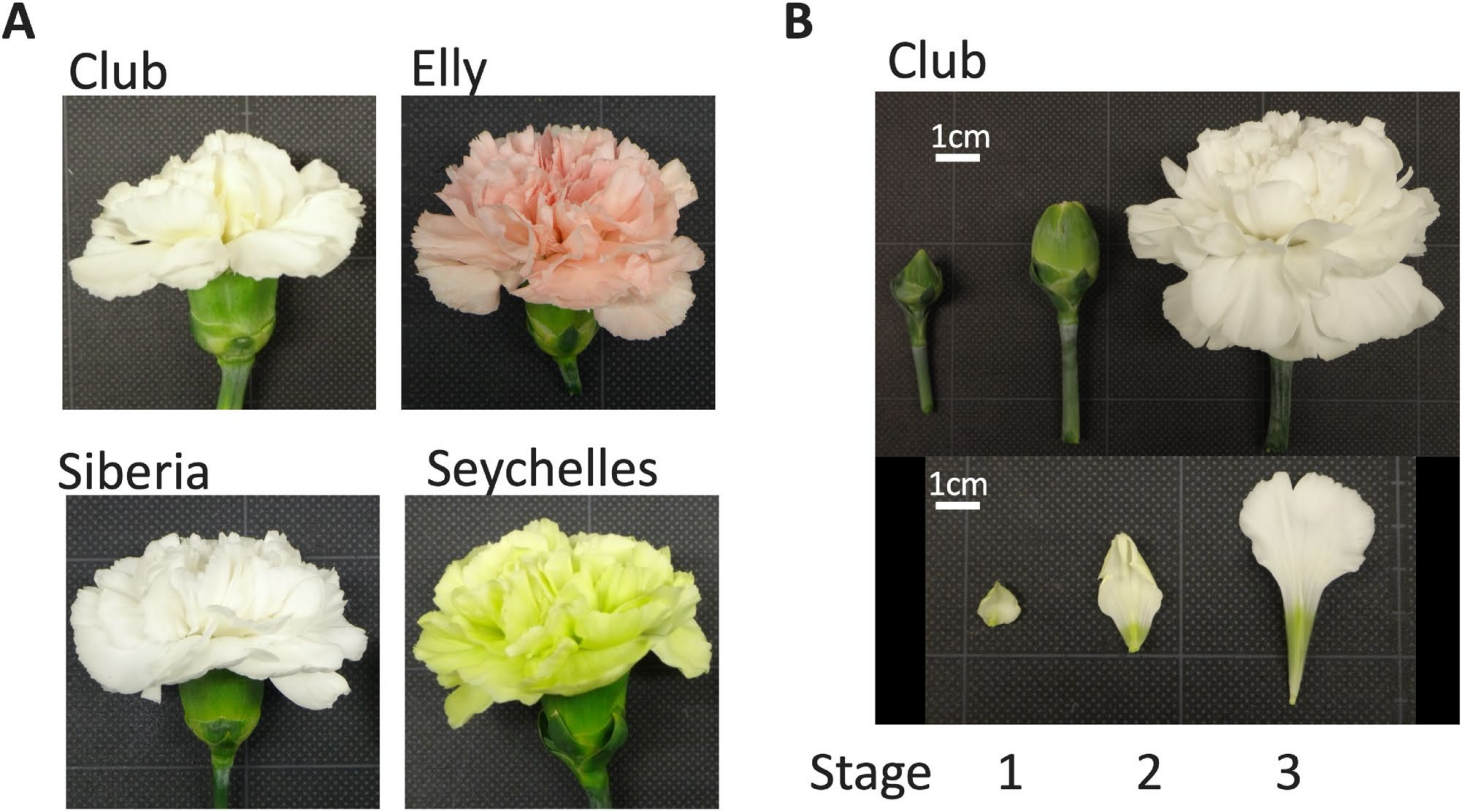
Photographs of carnation flowers used in this study. (**A**) Flowers of four carnation cultivars. (**B**) Flower developmental stages of ‘Club’. Stage 1: closed flower buds; stage 2: just opened flower buds in which the top of the petals can just be seen; stage 3: fully-opened flowers. A detailed description of the criteria for distinguishing developmental stages is given in “Plant materials and chemicals” of the “Methods” section.

**Figure 2:**
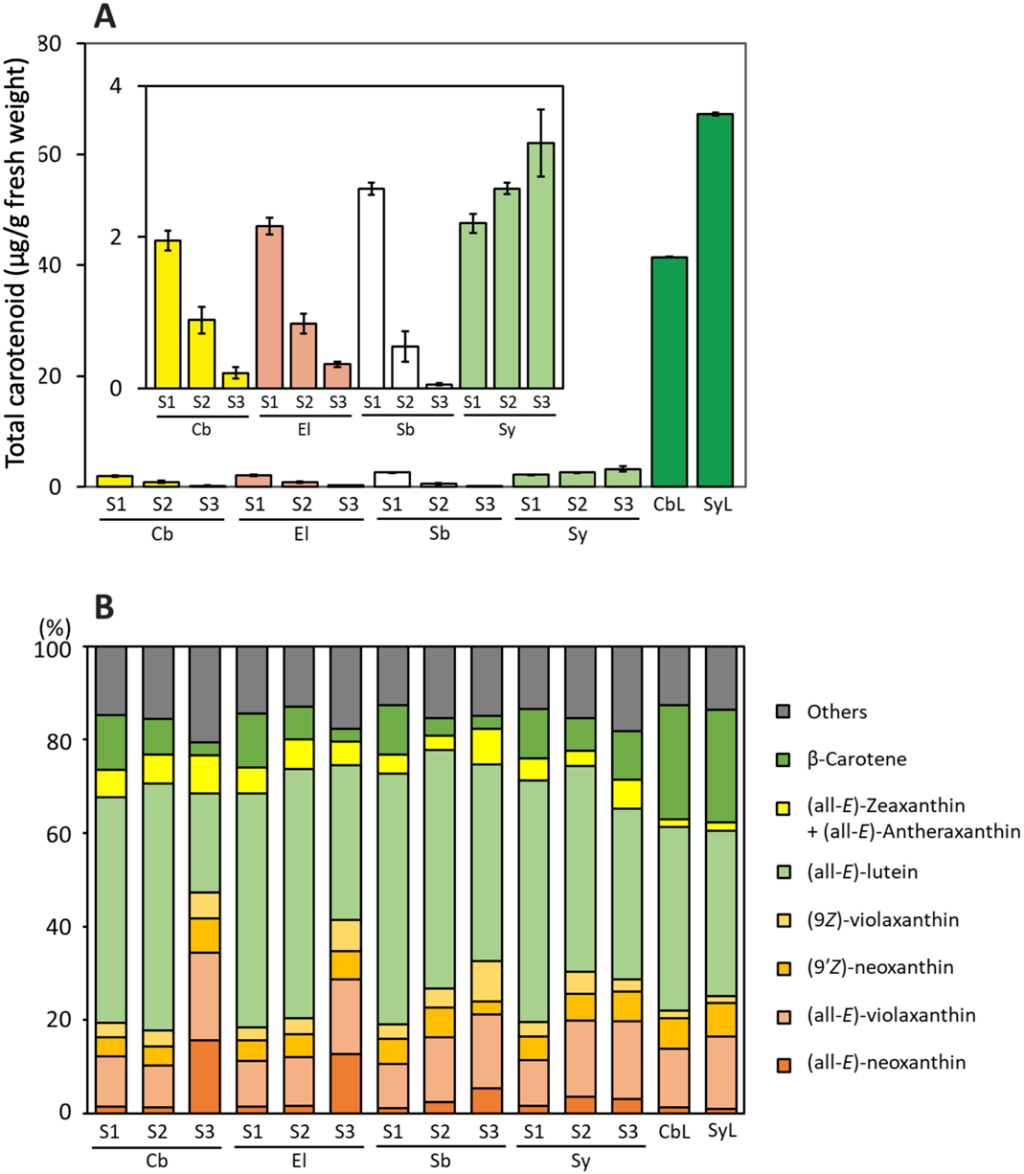
Changes in carotenoid content and composition in petals and leaves of carnations. Total carotenoid content (**A**) and carotenoid composition (**B**) in petals at different developmental stages and in mature leaves. Because the amounts of carotenoids were small compared to those in leaves, the vertical axis for the contents was magnified in the boxed insert in (**A**). Cb: ‘Club’; El: ‘Elly’; Sb: ‘Siberia’; Sy: ‘Seychelles’; S1, S2 and S3: petal developmental stages shown in Fig. 1B; CbL and SyL: mature leaves of ‘Club’ and ‘Seychelles’.

The pale yellow cultivar ‘Club’ was established from a bud mutant in the pink cultivar ‘Elly’ (Fig. 1). We performed an HPLC analysis of the carotenoid profiles in petals and leaves of ‘Club’, ‘Elly’, the white cultivar ‘Siberia’, and the green cultivar ‘Seychelles’. In all four cultivars, stage 1 petals had similar amounts of total carotenoids (Fig. 2A, Supplementary Figure S1). The carotenoid content of petals from ‘Club’, ‘Elly’, and ‘Siberia’ decreased during petal development; by contrast, carotenoid content in green petals of ‘Seychelles’ increased during development. Stage 1 and 2 petals of all cultivars were pale green; their carotenoid compositions were similar to those of leaves in which the majority of the carotenoids were (all-*E*)-lutein, β-carotene, and (all-*E*)-violaxanthin (Fig. 2B, Supplementary Figure S2). Carotenoid composition in petals of ‘Club’ and ‘Elly’ showed a considerable change at developmental stage 3; the relative content of (all-*E*)-neoxanthin increased, while that of (all-*E*)-lutein decreased. In the stage 3 petals of ‘Siberia’, the relative content of (all-*E*)-neoxanthin increased slightly, although the total carotenoid content was extremely low. In contrast, the carotenoid composition of petals of ‘Seychelles’ did not show much variation throughout petal development. The carotenoid composition of the pale green petals of ‘Seychelles’ was very similar to that of green leaves; the main carotenoids present were (all-*E*)-lutein, β-carotene, and (all-*E*)-violaxanthin; although the carotenoid compositions of petals and leaves were similar, the amount of β-carotene in leaves was 2.3 × higher than in petals at stage 3.

**Figure 3:**
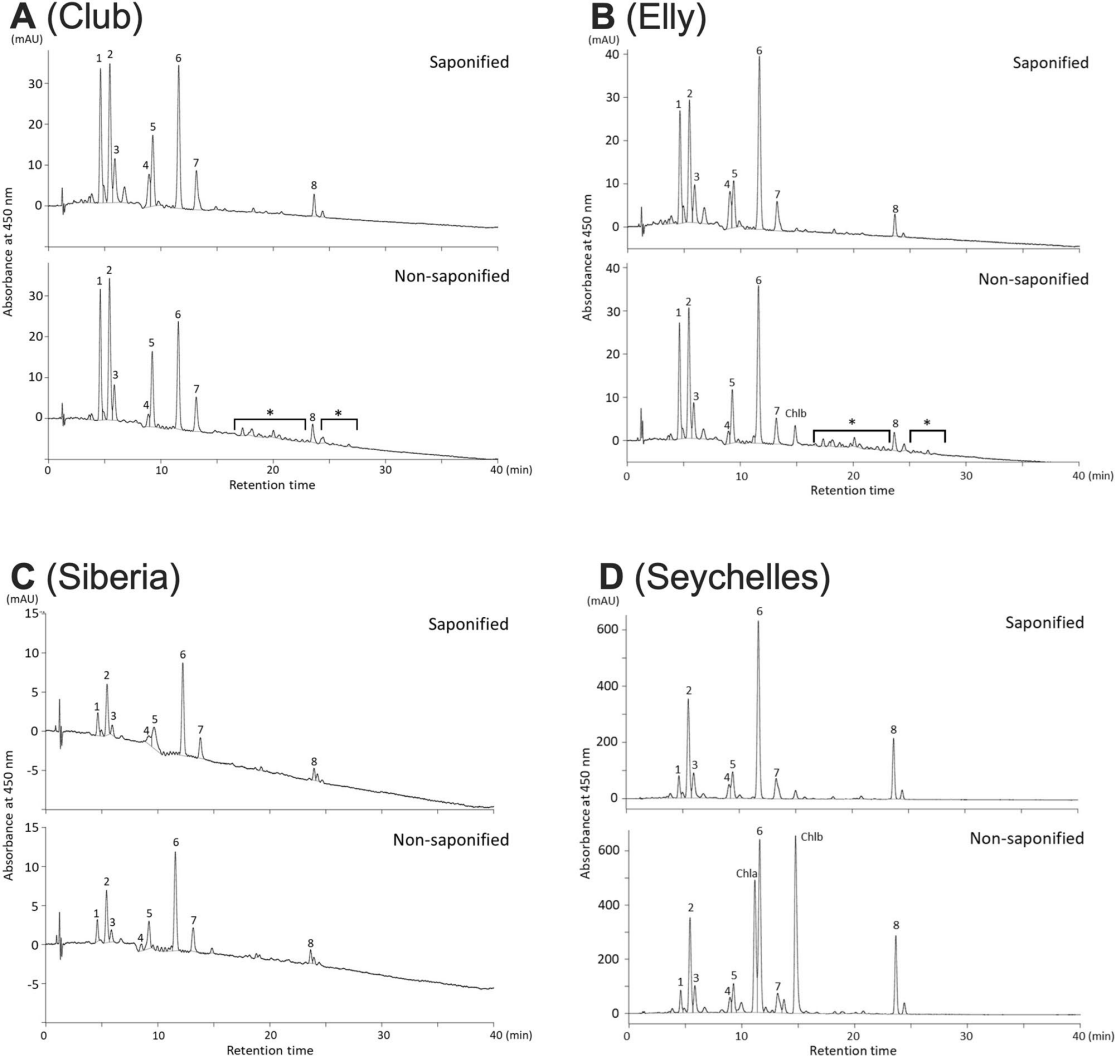
HPLC chromatograms of carotenoid extracts from petals at stage 3. (**A**) ‘Club’, (**B**) ‘Elly’, (**C**) ‘Siberia’ and (**D**) ‘Seychelles’. 1: (all-*E*)-neoxanthin; 2: (all-*E*)-violaxanthin; 3: (9′*Z*)-neoxanthin, 4: (9*Z*)-violaxanthin, 5: unknown; 6: (all-*E*)-lutein, 7: (all-*E*)-zeaxanthin + (all-*E*)-antheraxanthin, 8: β-carotene, Chla: chlorophyll *a*; Chlb: chlorophyll *b*; *: esterified xanthophylls. Chlorophylls found in non-saponified samples were degraded by the saponification procedure and not detected in the saponified HPLC analysis.

Comparison of HPLC chromatograms of saponified and non-saponified extracts showed that esterified xanthophylls were present in mature petals at stage 3 from ‘Club’ and ‘Elly’ (Fig. 3A,B, respectively). The levels of (9*Z*)-violaxanthin and (all-*E*)-lutein (peaks 4 and 6 in Fig. 3, respectively) increased in saponified extracts compared to non-saponified extracts, indicating some of the xanthophylls were esterified. In contrast, HPLC profiles of saponified and non-saponified samples in petals of ‘Siberia’ and ‘Seychelles’ were almost identical except for the presence of peaks for chlorophyll *a* and *b* in the non-saponified samples of ‘Seychelles’ (Fig. 3C,D). Esterified xanthophylls were not detected in HPLC chromatograms of mature leaves of ‘Club’ and ‘Seychelles’ (Supplementary Figure S3). We calculated the relative amounts of esterified to total xanthophylls by comparing the peak areas of each xanthophyll of saponified and non-saponified extracts (Table 1). More than 15% of total xanthophylls in the petals of ‘Club’ and ‘Elly’ were esterified. The proportions of esterified compounds in petals of ‘Club’ and ‘Elly’ were significantly higher (*P* < 0.05) than those of ‘Seychelles’. Although the contents of esterified xanthophylls in petals of ‘Elly’ were similar to those of ‘Seychelles’, the proportion of esterified compounds in ‘Elly’ was more than 10× higher than in ‘Seychelles’. The total content of xanthophylls in ‘Seychelles’ was almost 10× higher than in ‘Club’ and ‘Elly’. In petals of ‘Siberia’, the total xanthophyll content was approximately one-fourth and one-sixth of that of ‘Club’ and ‘Elly’, respectively; esterified xanthophylls were present at a very low level. The leaves of ‘Club’ and ‘Seychelles’ accumulated approximately 203.8× and 20.7× more total carotenoids, respectively, as the petals; no esterified xanthophylls could be detected.

**Table 1:** Contents and proportions of esterified and non-esterified xanthophylls in stage 3 petals and in mature leaves.

Tissue	Cultivar	Xanthophylls			Total carotenoid content (µg/g fresh weight)
		Esterification percentage (%)^†^	Esterified xanthophylls (µg/g fresh weight)	Total xanthophylls (µg/g fresh weight)	
Petals	Club	16.85 ± 3.56^a^^‡^	0.01999 ± 0.00238^b^^§^	0.1559 ± 0.0586^c^^¶^	0.203 ± 0.0762^c^^#^
	Elly	15.72 ± 2.62^a^^‡^	0.0387 ± 0.00686^a^^§^	0.253 ± 0.0304^c^^*¶*^	0.318 ± 0.0351^c^^#^
	Siberia	8.09 ± 1.658^ab^^‡^	0.00287 ± 0.000494^bc^^§^	0.0421 ± 0.01093^c^^*¶*^	0.0512 ± 0.01472^c^^*#*^
	Seychelles	1.335 ± 0.0209^b^^‡^	0.031 ± 0.00455^ab^^§^	2.32 ± 0.338^c^^*¶*^	3.25 ± 0.444^c^^#^
Leaves	Club	N.D.	N.D.	26.1 ± 1.807^b^^*¶*^	41.5 ± 2.77^b^^#^
	Seychelles	N.D.	N.D.	41.9 ± 0.0474^a^^*¶*^	67.3 ± 8.96^a^^#^

The data were collected from three biological replicates and presented as means ± standard errors (n = 3).

^†^ Proportion (%) of total xanthophyllys that were esterified.

^‡^,^§,¶,#^The different letters, "a", "b" and "c", in individual columns with the same symbol indicate significant differences among cultivars and tissues by Tukey’s test (*P* < 0.05) .

N.D., not detected.

### Expression of genes for carotenoid metabolism in petals

**Figure 4:**
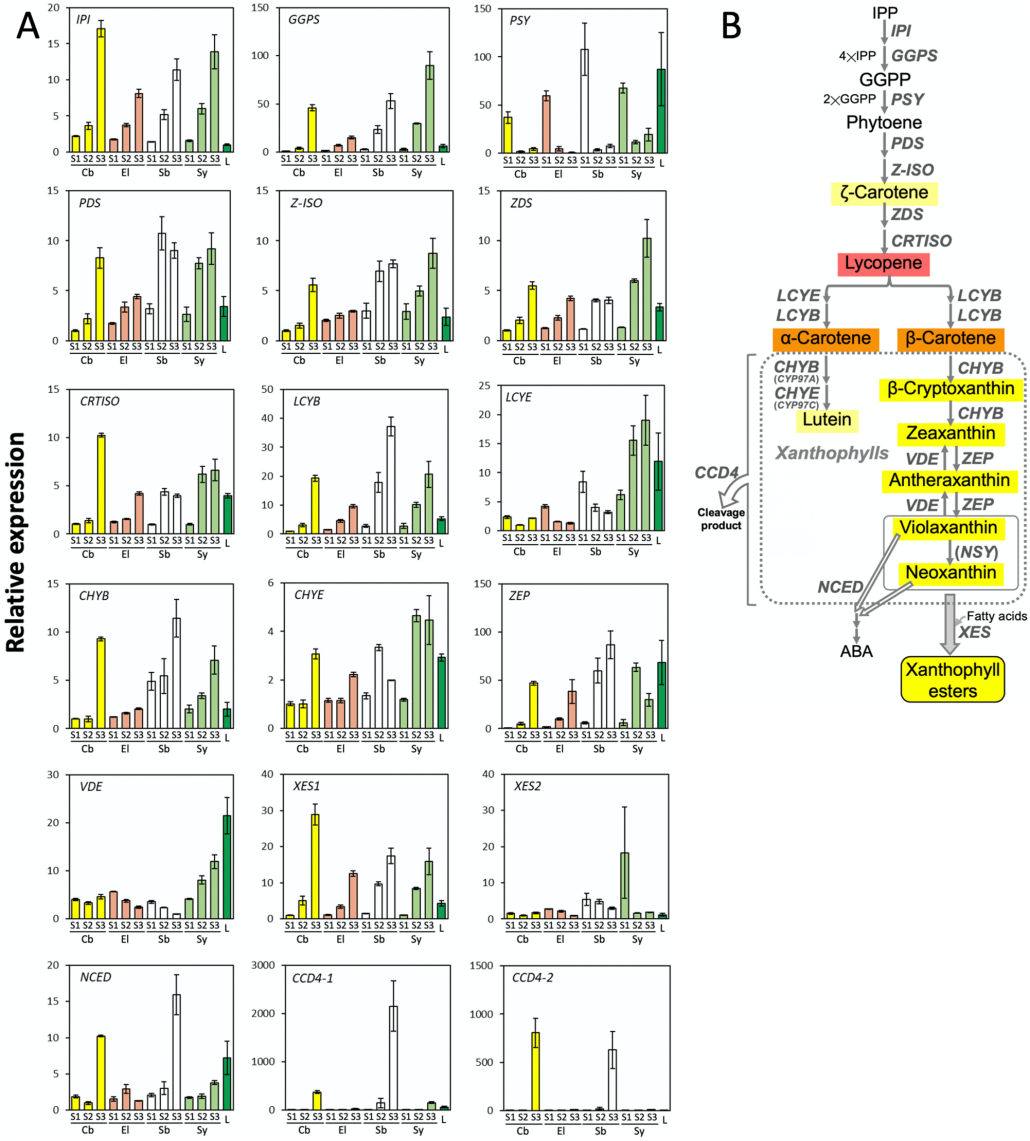
Expression profiles of genes encoding enzymes involved in carotenoid metabolism in carnation petals at different developmental stages. (**A**) Yellow, red, white, and pale green bars indicate the relative levels of gene expression in petals of ‘Club’, ‘Elly’, ‘Siberia’ and ‘Seychelles’, respectively, at stage 1, 2 and 3, which were calculated from the lowest ΔCt value after normalization against *actin*. Dark green bars on ‘L’ show expression levels in leaves of ‘Club’. (**B**) Metabolic pathway of carotenoids. IPP: isopentenyl diphosphate; IPI: IPP isomerase; GGPP: geranylgeranyl diphosphate; GGPS: GGPP synthase; PSY: phytoene synthase; PDS: phytoene desaturase; Z-ISO: ζ-carotene isomerase; ZDS: ζ-carotene desaturase; CRTISO: carotenoid isomerase; LCYB: lycopene β-ring cyclase; LCYE: lycopene ε-ring cyclase; CHYB: β-ring hydroxylase; CHYB/CYP97A: cytochrome P-450 type β-ring hydroxylase; CHYE: cytochrome P-450 type ε-ring hydroxylase; ZEP: zeaxanthin epoxidase; VDE: violaxanthin de-epoxidase; NCED: 9-*cis*-epoxy carotenoid dioxygenase; XES: xanthophyll esterase; CCD4: carotenoid cleavage dioxygenase 4; ABA: abscisic acid.

The patterns of expression of genes for enzymes involved in carotenoid synthesis and degradation were analyzed by RNA-seq analysis (Supplementary Figure S4) and by qRT-PCR (Fig. 4). Although slight differences in the estimated expression levels were found between the two methods, overall, they gave similar results for gene expression at Stage 3. Heatmaps prepared from the RNA-seq data (Supplementary Figure S4) provide an overview of gene expression profiles in the petals of the four cultivars at stage 3. The qRT-PCR data (Fig. 4A) describes the expression profile in more detail and can be used to identify changes in gene expression in petals during development.

We found increased expression of genes for enzymes involved in the carotenoid synthesis pathway from *isopentenyl diphosphate isomerase* (*IPI*) to *zeaxanthin epoxidase* (*ZEP*), with the exception of *PSY* and *LCYE*; this pattern of expression was seen in petals of all four cultivars during petal development (Fig. 4A). Neoxanthin was synthesized and found to accumulate (Fig. 2B); however, we failed to detect an annotated sequence for *neoxanthin synthase* (*NSY*) in the Carnation genome database (Carnation DB, https://carnation.kazusa.or.jp/)^35^. Therefore, we could not analyze expression of the gene by either RNA-seq or qRT-PCR. The expression of the *IPI* and *geranylgeranyl diphosphate synthase* (*GGPS*) genes that encode enzymes involved in synthesis of the first C40 carotenoid backbone, phytoene, were considerably up-regulated in stage 3 petals and their relative levels of expression were greater than in leaves. *PSY* showed high expression in stage 1 petals of all four cultivars, although its expression decreased thereafter (Fig. 4A).

Comparison of the four cultivars indicated that stage 3 petals of ‘Seychelles’ generally showed the highest levels of expression of genes encoding enzymes involved in carotenoid biosynthesis (Fig. 4A; Supplementary Figure S4A); this pattern of gene expression was consistent with the higher level of total carotenoids in ‘Seychelles’ (Table 1). The transcription levels of *PSY* and *LCYE* were much higher in petals of ‘Seychelles’ than in other cultivars.

RNA-seq analysis showed a high level of expression of most genes encoding enzymes involved in carotenoid degradation, including *CCD4*, *9-*cis*-epoxy carotenoid dioxygenase* (*NCED*), and *CCD1* in petals, in stage 3 petals of ‘Siberia’ (Supplementary Figure S4B). The qRT-PCR analysis showed that *CCD4-1* expression was more than 5× as high in stage 3 petals of ‘Siberia’ than in other cultivars (Fig. 4). The expression level of *NCED* was also highest in ‘Siberia’ petals among the four cultivars tested. However, the level of *CCD4-2* expression in petals of ‘Club’ was found to be high by qRT-PCR analysis, whereas it was very low by RNA-seq.

Although the estimated levels of *XES1* and *XES2* expression were different between qRT-PCR and RNA-seq analyses for stage 3 petals (Fig. 4A and Supplemental Figure S4B, respectively), *XES* expression did not seem to directly correlate with the levels of esterification in the four cultivars (Table 1). *XES1* expression, as measured by qRT-PCR, increased during petal developmental stages in all four cultivars. In the petals of ‘Club’, *XES1* expression was approximately twice as high at stage 3 than in the other cultivars; expression in ‘Elly’ petals was similar to that in ‘Siberia’ and ‘Seychelles’ petals. A high level of *XES2* expression was found in the stage 1 petals of ‘Seychelles’. In Carnation DB, the exon–intron junctions of *XES1* (Dca36220.1) were mis-predicted by the computer program and 11 exons were shown; however, when the mis-prediction was revised (Supplemental Table S1), *XES1* (Dca36220.1) consisted of 13 exons, the same as *XES2* (Dca11806.1). The revised XES1 (Dca36220.1) and XES2 (Dca11806.1) had 77.5% identity in nucleotide sequences and 72.0% identity and 92.4% similarity in amino acid sequences.

### Gene expression related to flower color phenotypes in carnation

Our previous reports showed that yellow and red petal colors in carnations are produced by the flavonoid chalcone 2′-*O*-glucoside and anthocyanins, respectively, while green is produced by chlorophyll^6,29–31^. Petals of all the cultivars used in this study synthesized flavonoid derivatives (Supplemental Figure S5). The major flavonoids detected here were flavonol glycosides, which produce colorless petals, as previously reported^3,30,31^. In petals of ‘Club’ and ‘Siberia’, chalcone 2′-*O*-glucoside was under the detectable level. The pink color of ‘Elly’ was generated by anthocyanins (Supplementary Figure S5) and the green color in ‘Seychelles’ came from chlorophylls (Fig. 3D); in both cultivars, a low level of chalcone 2′-*O*-glucoside was detected, but its effect on coloration was masked by the more abundant anthocyanins and chlorophylls.

Expression profiles of genes encoding enzymes involved in flavonoid and chlorophyll metabolism were analyzed by RNA-seq and the results shown as heatmaps (Supplementary Figure S6 and S7, respectively). Flavonoid metabolism, which follows phenylpropanoid metabolism, includes early biosynthetic genes (EBGs) that produce flavonols and late biosynthetic genes (LBGs) that produce anthocyanins^36^. In ‘Elly’, high expression of EBGs and LBGs were observed, resulting in synthesis and accumulation of anthocyanins in pink colored petals (Supplementary Figure S6). In ‘Seychelles’, genes for enzymes involved in phenylpropanoid metabolism and EBGs were highly expressed.

We analyzed the expression of genes involved in chlorophyll biosynthesis, the chlorophyll cycle, and chlorophyll degradation (Supplementary Figure S7). The petals of ‘Seychelles’ showed remarkably high levels of expression of genes for chlorophyll biosynthesis, whereas petals of ‘Siberia’ showed high levels of chlorophyll degradation compared to other cultivars. In ‘Club’ and ‘Elly’, extremely low levels of expression of all genes related to chlorophyll biosynthesis, the chlorophyll cycle, and chlorophyll degradation were found. The results suggested that chlorophyll biosynthesis activity in petals of ‘Seychelles’ was higher than those of other cultivars tested here, which caused higher level of chlorophyll accumulation.

### Ultrastructural analysis of petal plastids

**Figure 5:**
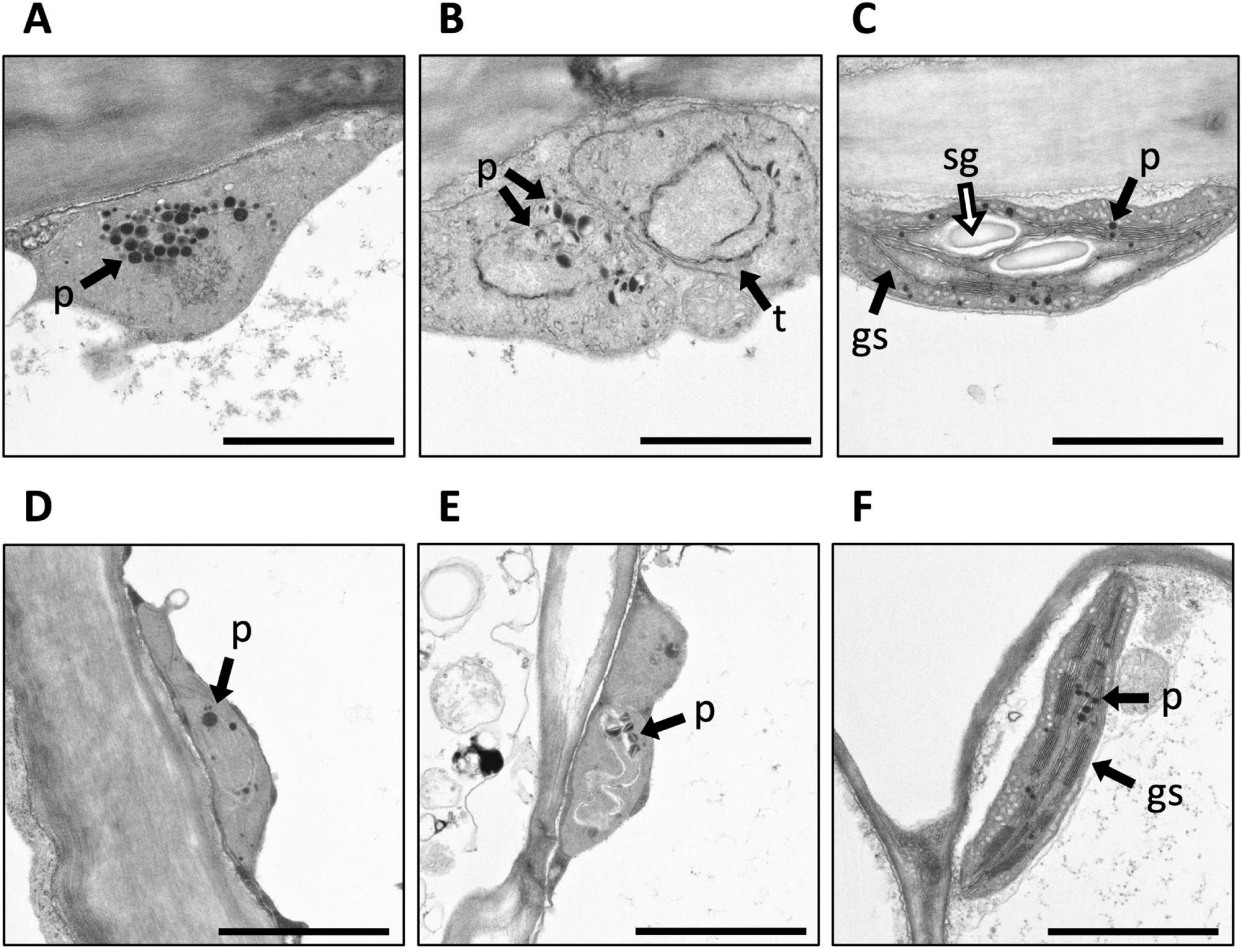
Comparison of plastid ultrastructure in petal cells among the three carnation cultivars. TEM analysis of plastid morphology in stage 3 petals of ‘Club’, ‘Siberia’ and ‘Seychelles’ plants. Plastids from epidermal cells of ‘Club’ (**A**), ‘Siberia’ (**B**), and ‘Seychelles’ (**C**), and from mesophyll cells of ‘Club’ (**D**), ‘Siberia’ (**E**), and ‘Seychelles’ (**F**). p, plastoglobules; t, thylakoid; gs, granal stacks; sg, starch grain. Bars = 1.0 μm.

As carotenoids are synthesized and accumulated in chromoplasts, we investigated plastid morphology in ‘Club’, ‘Siberia’, and ‘Seychelles’ using transmission electron microscopy (TEM). In petal epidermal cells of ‘Club’, a large number of plastids was observed with numerous plastoglobules (Fig. 5A); plastids in the petal mesophyll cells contained fewer and more immature plastoglobules (Fig. 5D). In ‘Siberia’, plastids in both epidermal and mesophyll cells (Fig. 5B,E, respectively) contained irregularly shaped plastoglobules and disorganized and electron-lucent membrane structures. These structures resembled the leucoplasts that are present in the white petals of Arabidopsis^37^. Cells from ‘Seychelles’ had a chloroplastic structure with a few very small plastoglobules, structured granal stacks, and several starch grains (Fig. 5C,F).

## Discussion

In plants of the order Caryophyllales, which includes carnations, yellow petals colored by carotenoids have not previously been reported. In this study, our analysis of carotenoids showed that petals of the carnation cultivars ‘Club’ and ‘Elly’ synthesize and accumulate esterified carotenoids, as is the case for yellow flowers of many other plant species^15,17,27–29,38^.

In carnation cultivars, yellow flower coloration results from chalcone 2′-*O*-glucoside, while cream is produced by flavonols^30–33^. Flavonoid synthesis and accumulation were detected in the petals of the four cultivars analyzed here; however, the level of chalcone 2′-*O*-glucoside was too low to be detected in ‘Club’ and ‘Siberia’. Petals of ‘Siberia’ contained the flavonoids kaempferol 3-*O*-sophoroside and kaempferol 3-*O*-glucosyl-(1 → 2)-[rhamnosyl-(1 → 6)-glucoside], similarly to the ‘White Sim’ cultivar that has white flowers^3,31^. ‘Club’, ‘Elly’, and ‘Seychelles’ were found to contain kaempferol 3-*O*-neohesperidoside as in ‘Bridal White’^30^, indicating that flavonols are not responsible for yellow petal coloration in ‘Club’. ‘Elly’ and ‘Seychelles’ have pink and green petals due to the accumulation of anthocyanins (Supplementary Figure S5) and chlorophylls (Fig. 3D), respectively, that mask the yellow color of the carotenoids. Our carotenoid analysis showed that petals of ‘Club’ accumulated much higher amounts of carotenoids than petals of ‘Siberia’. Hence, the results indicate that the yellowish coloration of ‘Club’ flowers is derived from carotenoids and not from flavonols or chalcone 2′-*O*-glucoside. This is the first report to provide evidence that yellow petal color in carnations can be derived from carotenoids.

In many plant species, the carotenoids synthesized and accumulated in immature petals are almost identical to those found in green tissues such as leaves and stems. Leaf-specific carotenoids are referred to as “chloroplast-type carotenoids” based on the plastid structure where they accumulate^38^. During petal development, chloroplasts differentiate into chromoplasts and, in accordance with this differentiation process, chlorophylls are degraded and the amount and composition of carotenoids form unique profiles depending on plant species and variety^39,40^. These petal-specific carotenoids are referred to as “chromoplast-type carotenoids”^38^. For example, there is a considerable increase in the relative amounts of (9*Z*)‐lutein and (9′*Z*)‐lutein‐5,6‐epoxide in chrysanthemums and of β-cryptoxanthin in *Ipomoea* sp. in mature petals^20,38^. In petals of ‘Club’ and ‘Elly’, stage 1 petals had a similar carotenoid composition to leaves; there was a large increase in the proportions of (all-*E*)-neoxanthin and a decrease in the proportion of (all-*E*)-lutein at stage 3. In contrast, the carotenoid composition of ‘Seychelles’ petals did not change during the course of petal development. These results imply that there is a transition in carotenoid composition from chloroplast-type to chromoplast-type in petals of ‘Club’ and ‘Elly’, whereas no transition occurs in petals of ‘Seychelles’.

The total amount of carotenoids accumulated in petals of ‘Seychelles’ was significantly higher than in the other cultivars analyzed here. Our qRT-PCR analysis indicated that *PSY* and *LCYE* expression was higher in stage 3 petals of ‘Seychelles’ than in other cultivars; this increased level of expression might be responsible for the relatively high amount of carotenoids in ‘Seychelles’. This conclusion is consistent with the outcome of a previous study^29,34^ in which it was found that most carotenogenic genes were expressed in green petals of ‘Seychelles’ at similar levels to the leaves, except for *IPI*, *GGPS*, *PSY1* and *LCYE* in stage 3 petals. Our RNA-seq analysis here found that the expression levels of *PSY* and *LCYE* in stage 3 white petals of ‘Siberia’ were considerably lower than those in leaves and green petals of ‘Seychelles’. PSY and LCYE have been reported to be key regulatory enzymes for carotenoid biosynthesis in other plant species^18,37,41^. On this basis, we assume that PSY and LCYE are also key enzymes for carotenoid biosynthesis in carnation petals. A low level of these key enzymes at late stages of petal development might reduce the rate of carotenoid biosynthesis in petals of ‘Club’, ‘Elly’, and ‘Siberia’.

The patterns of gene expression for carotenoid biosynthesis in stage 3 petals of ‘Siberia’ were similar to those of ‘Club’ and ‘Elly’ (Fig. 4A), although total carotenoid content was very low (Table 1). In petals of ‘Siberia’, the expression of *NCED*s and *CCD4*s was much higher than in other cultivars (Supplementary Figure S4B). It has been reported for other plant species that CCD4 cleaves synthesized carotenoids and is responsible for the absence of carotenoids in petals^8,22–26^. In chrysanthemum petals, there is a reciprocal relationship between the level of *CCD4* transcript and carotenoid content^22,42^. These observations indicate that ‘Siberia’ petals has higher carotenoid degradation activity leading to extremely low levels of carotenoids. The results suggest that selection of carnation lines showing low *CCD4* expression or its absence is essential for breeding cultivars that have deep yellow flowers through the presence of carotenoids.

The relative amounts of esterified xanthophyll molecules in ‘Club’ and ‘Elly’ petals were significantly greater than in ‘Seychelles’ petals (Table 1). However, our qRT-PCR analysis showed similar levels of *XES* expression in petals of all four cultivars (Fig. 4). Ariizumi et al.^27^ showed that disruption of the *PALE YELLOW PETAL 1* (*PYP1*) gene that encodes an XES enzyme caused loss of esterified xanthophylls and produced a significant decrease in total carotenoid content. In addition, Kishimoto et al.^28^ showed that more than 80% of total xanthophylls exist in the esterified form in deep yellow petals of calibrachoa, and that this rate of esterification is associated with a high level of *XES* expression. The rates of esterification in petals of ‘Club’ and ‘Elly’ were much lower than in calibrachoa. These observations suggest that carnation flowers with a deeper yellow color due to the accumulation of more esterified xanthophylls might be obtained by selecting carnation lines with higher levels of expression of *XES* genes.

In fruits and flowers, carotenoids accumulate in the plastoglobules of chromoplasts, and are responsible for yellow to red colors^9,29^. Carotenoid sequestration and accumulation into plastoglobules is associated with plastid differentiation from chloroplast to chromoplast during petal development. In general, yellow petals tend to increase the number and size of chromoplasts and increase the number of plastoglobules during petal development, leading to an increase in carotenoid content^9^. The presence of chromoplasts in ‘Club’ petals was confirmed by our ultrastructural analysis; however, plastoglobule structures showing high electron-dense particles were observed in epidermal cells of mature petals (Fig. 5A). In wild-type tomato flowers, electron-dense immature plastoglobules were observed at an early stage of development; the electron density of plastoglobules then decreased as esterified xanthophylls accumulated^27^. In *pyp1* mutants of tomato, the electron density of plastoglobules is high throughout petal development and the chromoplast structure shows disruption at later developmental stages. We assume that the high electron-dense particles in plastoglobules of ‘Club’ might reflect a low rate of esterified xanthophylls caused by low expression of *XES.*

The present study is the first to show that carnation cultivars can synthesize and accumulate esterified carotenoids in their petals. It is still possible that as yet unidentified carnation cultivars may have a petal color derived from carotenoids that is masked by the green, yellow, and red colors of chlorophylls, flavonoids, and anthocyanins, respectively. It was previously believed that orange coloration in carnation flowers was generated by co-accumulation of red anthocyanin and yellow chalcone 2′-*O*-glucoside^6^. However, it is possible that orange-colored carnations with a combination of red anthocyanins and yellow carotenoids have been overlooked. In this study, we showed that carnations can synthesize and accumulate carotenoids in the chromoplasts although the levels are generally low. To enhance carotenoid accumulation in carnation petals, it will be necessary to increase the rate of biosynthesis and esterification of carotenoids. Further research will be needed to elucidate the mechanism that promotes carotenoid biosynthesis and esterification to establish much deeper yellow-flowered carnations as novel cultivars.

## Methods

### Plant materials and chemicals

The carnation (*Dianthus caryophyllus* L.) cultivars ‘Club’ (yellow) and ‘Elly’ (pink) were obtained from Barberet and Blanc S.A. (Puerto Lumbreras, Spain), and ‘Siberia’ (white) and ‘Seychelles’ (green) were gifts from Inochio Fujiplants Inc. (Aichi, Japan). Carnation plants were grown in a greenhouse at the National Agriculture and Food Research Organization (Tsukuba, Ibaraki, Japan). As the timing of flower growth differs among cultivars, petal development stages were visually defined as follows: stage 1 (Fig. 1B), immature petals in tightly closed buds that had a faint pale green coloration in all cultivars; additionally, the petals did not show yellow coloration in ‘Club’ nor were they tinged with pink in ‘Elly’; stage 2 (Fig. 1B), petals in just opened buds, with pale yellow petals in ‘Club’ and faint pink petals in ‘Elly’; stage 3, petals of fully-opened flowers when the outermost petals were flat or slightly downward tilted, as shown in Fig. 1A,B^3,5^. Petals and mature leaves were harvested and immediately frozen in liquid nitrogen, and stored at −80 °C until use.

All chemical reagents used in the experiments were SAJ special grade or HPLC grade from Sigma-Aldrich (St Louis, MO, USA), nacalai tesque Inc. (Kyoto, Japan), and FUJIFILM Wako Pure Chemical Co. (Osaka, Japan). The standard molecules for carotenoids were obtained commercially: (all-*E*)-violaxanthin from DHI, Hoersholm, Denmark; (9′*Z*)-neoxanthin from DHI; (all-*E*)-lutein from Sigma-Aldrich; (all-*E*)-zeaxanthin from Extrasynthese, Lyon-Nord, France; and β-carotene from Sigma-Aldrich. The authentic standard for chalcone 2′-*O*-glucoside was a gift from Dr. Iwashina and Dr. Mizuno, National Museum of Nature and Science (Tsukuba, Ibaraki, Japan).

### Extraction and analysis of carotenoids

Samples of petals (1 g) and leaves (0.1 g) were ground into fine powder in liquid nitrogen using a homogenizer. The powder was thawed and extracted using 2 mL of acetone and 100 μL of 1 M Tris–HCl buffer (pH 8.0) using vigorous vortexing. The mixture was partitioned by addition of 3 mL diethyl ether with vigorous mixing. The organic layer was recovered and 3 mL diethyl ether was added to the water phase for re-extraction. The extracted organic layers were combined and washed with 5 mL of 5 mM Tris–HCl buffer (pH 8.0), and the organic layer was divided into two parts to prepare non-saponified and saponified samples. Saponification was performed using an equivalent volume of 5% KOH/methanol (MeOH) (w/v) for 1 h at room temperature. The non-saponified sample was dried and dissolved in MeOH. The KOH-treated sample was extracted with diethyl ether and washed with water. The organic layer was dried and dissolved in MeOH to give a saponified sample. The dried extract was dissolved in MeOH and analyzed by high performance liquid chromatography (HPLC) (X-LC, JASCO, Tokyo, Japan) under the following conditions: column, YMC Carotenoid (100 mm × 4.6 mm i.d., 3 μm; YMC, Kyoto, Japan); solvent A, MeOH/methyl *tert*-butyl ether (MTBE)/H_2_O = 95:1:4 (v:v:v) and solvent B, MeOH/MTBE/H_2_O = 25:71:4 (v:v:v). The gradient program was 0% B isocratic (5 min) and 0% B to 100% B (33 min) at a flow rate of 1.0 mL/min at 35 °C. Carotenoid peaks were detected at 450 nm (X-LC 3110MD, JASCO). Peaks were identified by comparing the retention times and absorbance spectra with those of carotenoids previously identified in the petals of chrysanthemum^43^, calendula (*Calendula officinalis*)^44^ and tomato^27^ and using commercially available standards for (all-*E*)-violaxanthin, (9′*Z*)-neoxanthin, (all-*E*)-lutein, (all-*E*)-zeaxanthin, and β-carotene. The amounts of individual and total carotenoids were estimated calculated as lutein equivalents from the peak areas of the HPLC chromatograms at 450 nm using the calibration curve of the lutein standard. The amount of esterified xanthophylls was calculated from the difference in peak areas between saponified and non-saponified samples. Measurements were performed in biological triplicates. The results were analyzed statistically using Tukey’s test for significant differences (*P* < 0.05).

### Extraction and analysis of flavonoids

Flavonoids and anthocyanins in 0.1 g samples of petals were extracted with 10 mL of 80% MeOH or 1 mL of 80% MeOH containing 0.1% trifluoroacetic acid, respectively, at 4 °C overnight. Debris was removed from extracts by centrifugation at 2,500×*g* for 10 min. After passing the supernatant through a membrane filter, flavonoids and anthocyanins in the extracts were analyzed by an ultraperformance liquid chromatography (UPLC)-Q Exactive hybrid quadrupole-orbitrap mass spectrometer (Thermo Fisher Scientific, Waltham, MA, USA) equipped with an Acquity UPLC HSS T3 column (100 mm × 2.1 mm i.d, 1.8 μm; Waters, Milford, MA, USA) under the same conditions as previously reported^45^ except for elution conditions: solvent A, 0.1% aqueous solution of formic acid and solvent B, acetonitrile containing 0.1% formic acid. The gradient program was 5% B to 40% B (8 min) and 40% to 95% B (4 min) followed by 95% B isocratic (4 min) at a flow rate of 0.4 mL/min at 40 °C. The UV–vis data were collected over the range 198–600 nm. The wavelength for detection was set to 350 nm for flavonoids, 410 nm for chalcones, and 510 nm for anthocyanins. Data processing was performed using Xcalibur software (Thermo Fisher Scientific).

### RNA-seq and data analysis

Total RNAs were extracted from petal tissues of individual stages 1 to 3 using the RNeasy Plant Mini Kit (Qiagen, Courtaboeuf, France) according to the manufacturer’s instructions. RNA was treated with RQ1 RNA-Free DNase (Promega KK, Tokyo, Japan) to remove contaminating genomic DNA. Construction of RNA-seq libraries and the protocol for the RNA-seq analysis using Illumina next generation sequencer have been reported previously^46^. Sequence raw data was registered in DNA Data Bank of Japan (DDBJ) under BioProject ID PRJDB9191. Nucleotide sequences from petals of each cultivar were trimmed of adaptor and low-quality sequence regions. All sequences were mapped to the predicted transcriptome sequences and annotation data of carnation cv. ‘Francesco’ genome (DCA_r1.0_cds.fna, downloaded from Carnation DB) and calculated transcripts per kilobase million (TPM) by salmon 0.14.1. Heatmaps were displayed using *z*-score normalized mean-centered log2 TPM using the packages gplots 3.0.1.1 and genefilter 1.68.0 in R. Dca numbers are gene ID annotated in Carnation DB^35^.

### Quantitative RT-PCR analysis

Total RNAs were obtained as described above from petals and leaves and first-strand cDNA was synthesized from 1 µg total RNA using PrimeScript Reverse Transcriptase (Takara Bio Inc., Shiga, Japan) and oligo (dT) primers. The transcript levels were analyzed using quantitative RT-PCR (qRT-PCR) with Thunderbird SYBR qPCR Mix (TOYOBO, Co. Ltd., Osaka, Japan) and a DNA Engine Opticone 2 system (Bio-Rad Laboratories, Hercules, CA, USA). The first strand cDNA reaction mixture was diluted three-fold with water and 1 µL of the diluted cDNA mixture was added to 10 μL 2 × SYBR qPCR Mix and 0.3 μM each of the forward and reverse gene specific primers shown in Supplementary Table S2. The reaction mixtures were heated to 95 °C for 1 min, followed by 35 cycles at 95 °C for 30 s, 58 °C for 30 s, and 72 °C for 30 s. The quantification of RT-PCR was performed by ΔCt. The expression level of *actin* was used to normalize the transcript levels of each gene. Relative expression of each sample was calculated from the sample with the lowest ΔCt value after normalization against *actin*. The assays were performed in biological triplicate.

### TEM imaging

Petals at stage 3 were cut into pieces of about 1 mm^3^, fixed, dehydrated, and embedded in Quetol 651 (Nisshin EM Co., Tokyo, Japan) as described previously^47^. Ultrathin sections were cut with a diamond knife on an ultramicrotome (Ultracut UCT, Leica Vienna, Austria) and the sections were mounted on copper grids, stained with uranyl acetate and lead citrate, and observed under a TEM (JEM-1400Plus; JEOL Ltd., Tokyo, Japan) at an acceleration voltage of 100 kV. Digital images were taken with a CCD camera (EM-14830RUBY2; JEOL).

## Supplementary information

Supplementary Information.
