# Supplementary material for: Esterified carotenoids are synthesized in petals of carnation (Dianthus caryophyllus) and accumulate in differentiated chromoplasts
Source: Sci Rep. 2020 Sep 16;10:15256. doi: 10.1038/s41598-020-72078-4 (PMC7495429; doi:10.1038/s41598-020-72078-4)
Supplement: Supplementary file 1 — Supplementary Information. [file 41598_2020_72078_MOESM1_ESM.pdf]

Supplementary Informations

Title: Esterified carotenoids are synthesized in petals of carnation (*Dianthus caryophyllus*) and accumulate in differentiated chromoplasts

Authors: Luna Iijima, Sanae Kishimoto, Akemi Ohmiya, Masafumi Yagi, Emi Okamoto, Taira Miyahara, Takashi Tsujimoto, Yoshihiro Ozeki, Nahoko Uchiyama, Takashi Hakamatsuka, Takanobu Kouno, Emilio A. Cano, Motoki Shimizu, Masahiro Nishihara

Supplementary Table S1. Revised exon-intron junctions of *XES1* (Dca35220.1) and *XES2* (Dca11806.1) genes

XES1	Dca36220.1			
**seqname**	**source**	**feature**	**start**	**end**
scaffold38	Augus-PASA	mRNA	386206	391238
scaffold38	Augus-PASA	exon 13	386206	386515
scaffold38	Augus-PASA	exon 12	386712	386797
scaffold38	Augus-PASA	exon 11	386918	387031
scaffold38	Augus-PASA	exon 10	387284	387644
scaffold38	revised	exon 9	387745	387878
scaffold38	revised	exon 8	388155	388263
scaffold38	Augustus	exon 7	388872	388993
scaffold38	Augustus	exon 6	389162	389268
scaffold38	Augustus	exon 5	389447	389551
scaffold38	Augustus	exon 4	389638	389796
scaffold38	revised	exon 3	390007	390062
scaffold38	revised	exon 2	390319	390370
scaffold38	Augustus	exon 1	390824	391238

XES2	Dca11806.1			
**seqname**	**source**	**feature**	**start**	**end**
scaffold153	Augustus	mRNA	256665	263713
scaffold153	Augustus	exon 13	256665	257058
scaffold153	Augustus	exon 12	257579	257630
scaffold153	Augustus	exon 11	258473	258528
scaffold153	Augustus	exon 10	259029	259187
scaffold153	Augustus	exon 9	259285	259389
scaffold153	Augustus	exon 8	259859	259965
scaffold153	Augustus	exon 7	260403	260524
scaffold153	Augustus	exon 6	260675	260783
scaffold153	Augustus	exon 5	261461	261594
scaffold153	Augustus	exon 4	261950	262310
scaffold153	Augustus	exon 3	262650	262763
scaffold153	Augustus	exon 2	263039	263124
scaffold153	Augustus	exon 1	263407	263713

Iijima *et al*. Supplementary Table S1

Supplementary Table S2. Primers used for RT-qPCR analysis.

gene		sequence	carnation DB ID	start position	end position
IPI	FW	5'-ACCAACACGTGCTGCAGTCA-3'	Dca51963.1	259	278
	RV	5'-CCTTAATGCCGAGCTCGTCT-3'		373	354
GGPS	FW	5'-CGCACAAGGTTTTCGGTGAG-3'	Dca29907.1	584	603
	RV	5'-TGCTCAAACGCAAACGACAG-3'		653	634
PSY	FW	5'-GGAATGAGAATGGATCTCCG-3'	Dca56296.1<br>Dca46314.1<br>Dca11863.1	682	701
	RV	5'-CTAATGCAGCATTGTAGACACT-3'		823	844
PDS	FW	5'-CTCGCTTGCAAAAGATTG-3'	Dca52111.1<br>Dca48369.1<br>Dca36978.1	1004<br>980<br>776	1021<br>997<br>793
	RV	5'-CGGGTACAAGCAACTTAAAG-3'		1116<br>1092<br>319	1135<br>1111<br>304
Z-ISO	FW	5'-ATAGGCCTGATTGGGCATCA-3'	Dca5563.1	832	851
	RV	5'-GGATAATGCTTGTACGATCT-3'		921	940
ZDS	FW	5'-AGGACATAGAAAAATCTCGG-3'	Dca28883.1	875	894
	RV	5'-CCTTGCCCTGGAATATAGTA-3'		991	1010
CRTISO	FW	5'-TGGAGAAATAGCTCGGAAA-3'	Dca1396.1	633	651
	RV	5'-GCCGATCACATAGTACCATA-3'		747	766
LCYB	FW	5'-ACGTTTCTGTATGCTATGCC-3'	Dca47589.1	847	866
	RV	5'-TCTTCGATGCTTTTAACCCT-3'		979	998
LCYE	FW	5'-TTAATCAAGGCTGGTCACTC-3'	Dca41710.1<br>Dca23076.1	1147<br>1672	1166<br>1691
	RV	5'-GAAGAACGCTCGCTGACGCT-3'		1247<br>1772	1266<br>1791
CHYB	FW	5'-GGCGAGGTGCCAGTTACGGA-3'	Dca61272.1	358	377
	RV	5'-GACTCGTGCATGTGCCACAA-3'		469	488
CHYE	FW	5'-ATTTAGTGGAGGTCCTCGTAA-3'	Dca53512.1<br>Dca35372.1	1209<br>1209	1229<br>1229
	RV	5'-CCCGTCGTCATAGTAATGTC-3'		1321<br>1321	1340<br>1340
ZEP	FW	5'-TGCTATGCAACCCAATATG-3'	Dca60413.1	1125	1143
	RV	5'-TCGCAGTTTTCTAGATCCTT-3'		1268	1287
VDE	FW	5'-CGAGTTCCATGTCGAAGATG-3'	Dca7401.1	726	745
	RV	5'-TTGCACAGCTGATCTCGTGA-3'		800	819
NCED	FW	5'-TACCACGTGAGGACGGAA-3'	Dca17005.1	1580	1597
	RV	5'-TGGAAACCGTATGGTACTCTG-3'		1716	1736
CCD4-1	FW	5'-ATCCGCAATGGTCCAAAC-3'	Dca37305.1	478	495
	RV	5'-TACGTCTTGACATAGCGGC-3'		590	608
CCD4-2	FW	5'-GACACTTTTGAATATGTTGG-3'	Dca57229.1	300	319
	RV	5'-CGGTTGGAGGAAGCTCAACA-3'		428	408
XES1	FW	5'-ATTTGTGCGAATGGCTGCGA-3'	Dca36220.1	1737	1756
	RV	5'-CTCATAAGATCATCGTAGTC-3'		1822	1841
XES2	FW	5'-ATACTTTCAAACCCTGCAACATCGA-3'	Dca11806.1	646	670
	RV	5'-ATTAACCGTCGCCATCTTCTTTCC-3'		772	795

Supplementary Figure S1. HPLC chromatograms of carotenoid extracts from stage 1 petals.

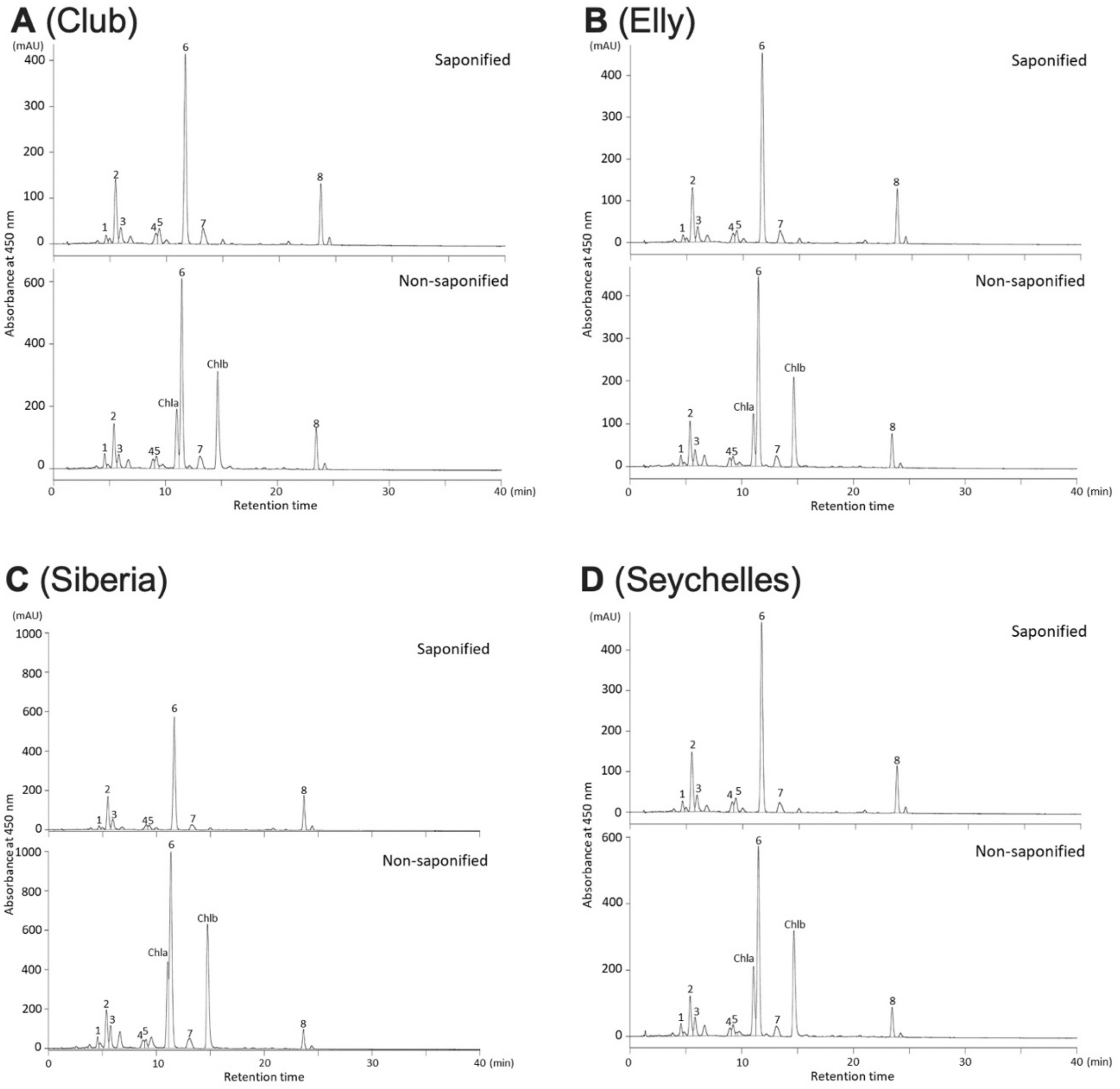

Supplementary Figure S1. HPLC chromatograms of carotenoid extracts from stage 1 petals.
(A) 'Club', (B) 'Elly', (C) 'Siberia' and (D) 'Seychelles'. 1: (all-*E*)-neoxanthin; 2: (all-*E*)-violaxanthin; 3: (9′*Z*)-neoxanthin, 4: (9*Z*)-violaxanthin, 5: unknown; 6: (all-*E*)-lutein, 7: (all-*E*)-zeaxanthin + (all-*E*)-antheraxanthin, 8: β-carotene, Chla: chlorophyll *a*; Chlb: chlorophyll *b*.

Supplementary Figure S2. HPLC chromatograms of carotenoid extract from 'Club' petals and commercial standards.

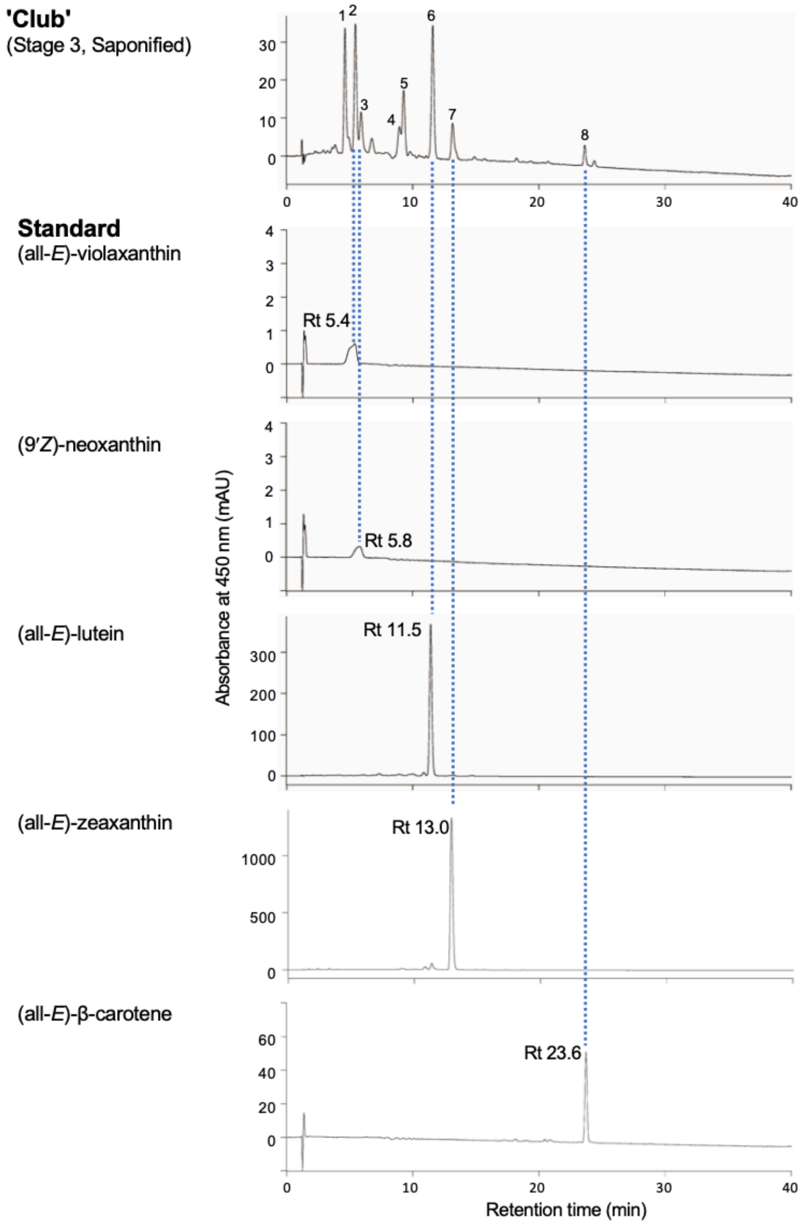

Supplementary Figure S2. HPLC chromatograms of carotenoid extract from 'Club' petals and commercial standards.

Peak 1: (all-*E*)-neoxanthin (Rt = 4.6 min at 'Club'); 2: (all-*E*)-violaxanthin (Rt = 5.5 min); 3: (9′Z)-neoxanthin (Rt = 5.9 min), 4: (9*Z*)-violaxanthin (Rt = 9.0 min), 5: unknown (Rt = 9.3 min); 6: (all-*E*)-lutein (Rt = 11.6 min), 7: (all-*E*)-zeaxanthin + (all-*E*)-antheraxanthin (Rt = 13.2 min), 8: (all-*E*)-β-carotene (Rt = 23.6 min).

Iijima *et al*. Supplementary Figure S2

Supplementary Figure S3. HPLC chromatograms of carotenoid extracts from mature leaves.

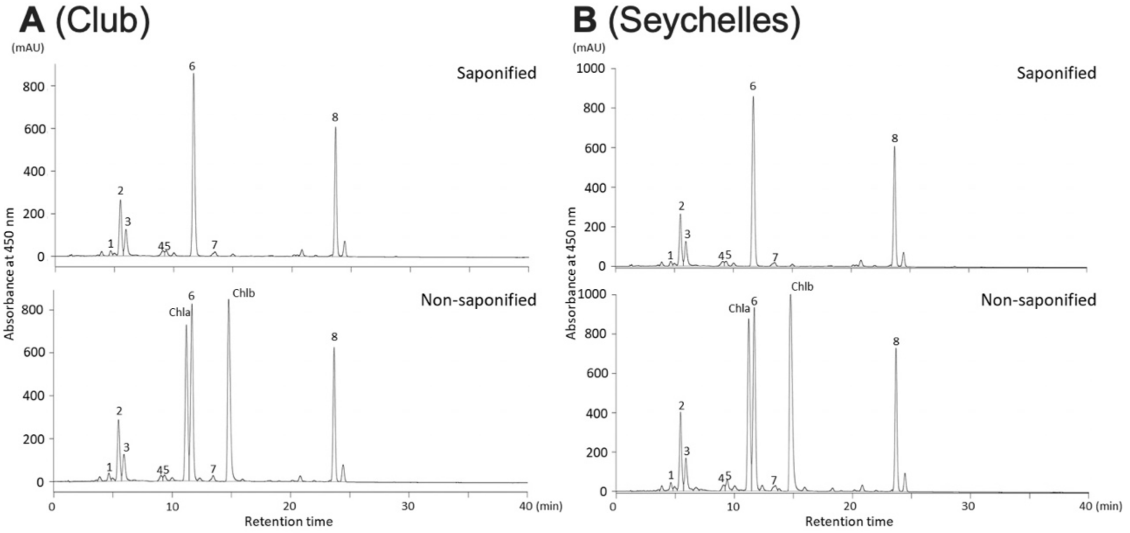

Supplementary Figure S3. HPLC chromatograms of carotenoid extracts from mature leaves.

(A) 'Club', (B) 'Seychelles'. 1: (all-*E*)-neoxanthin; 2: (all-*E*)-violaxanthin; 3: (9′*Z*)-neoxanthin, 4: (9*Z*)-violaxanthin, 5: unknown; 6: (all-*E*)-lutein, 7: (all-*E*)-zeaxanthin + (all-*E*)-antheraxanthin, 8: β-carotene, Chla: chlorophyll *a;* Chlb: chlorophyll *b.*

Supplementary Figure S4. Heatmaps of expression profiles of genes related to carotenoid synthesis (A) and degradation and modification (B) in stage 3 petals of four cultivars.

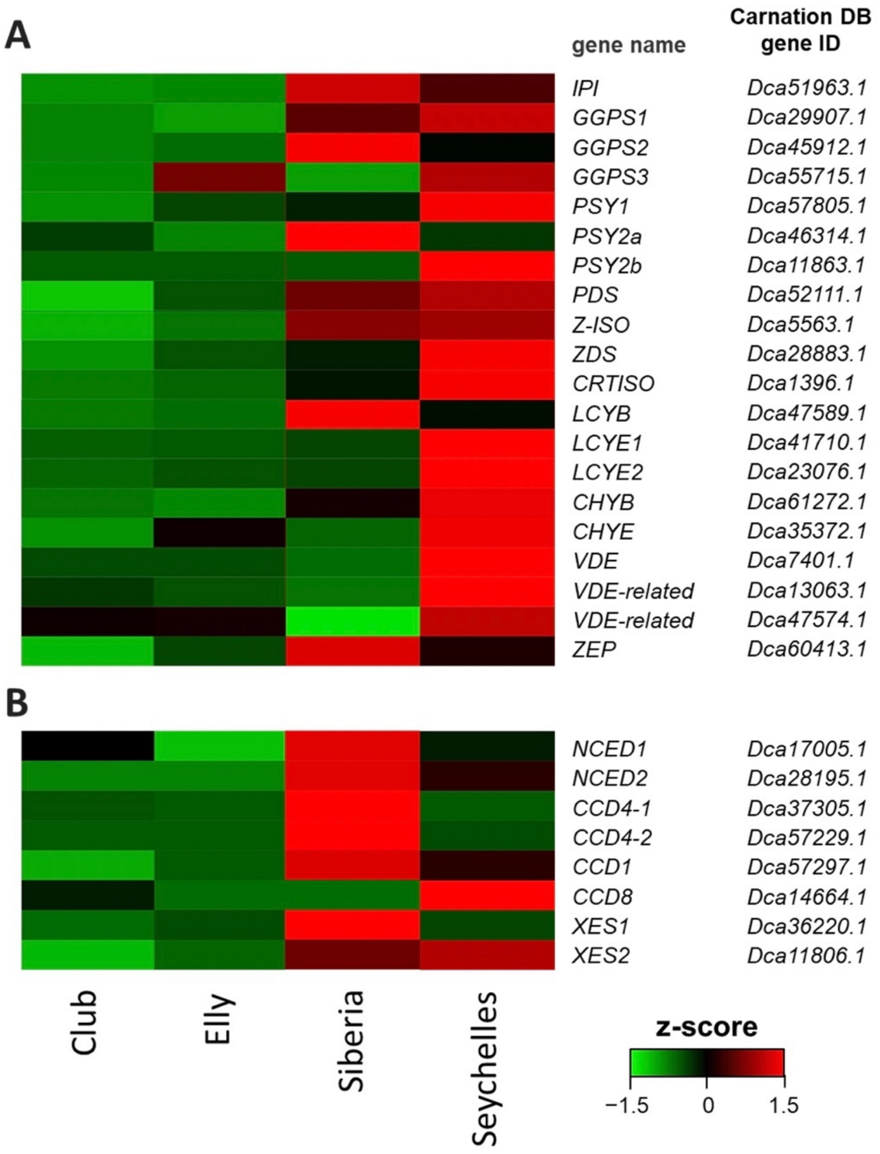

Supplementary Figure S4. Heatmaps of expression profiles of genes related to carotenoid synthesis (A) and degradation and modification (B) in stage petals of four cultivars.
IPP: isopentenyl diphosphate; IPI: IPP isomerase; GGPP: geranylgeranyl diphosphate; GGPS: GGPP synthase; PSY: phytoene synthase; PDS: phytoene desaturase; Z-ISO: ζ-carotene isomerase; ZDS: ζ-carotene desaturase; CRTISO: carotenoid isomerase; LCYB: lycopene β-ring cyclase; LCYE: lycopene ε-ring cyclase; CHYB: β-ring hydroxylase; CHYB/CYP97A: cytochrome P-450 type β-ring hydroxylase; CHYE: cytochrome P-450 type ε-ring hydroxylase; ZEP: zeaxanthin epoxidase; VDE: violaxanthin de-epoxidase; NCED: 9-*cis*-epoxy carotenoid dioxygenase; ABA: abscisic acid; XES: xanthophyll esterase; CCD4: carotenoid cleavage dioxygenase 4. The clustering was based on TPM values which were log transformed and z-score normalized. The *z*-score values are continuously mapped on the color key provided at the bottom of the figure. Dca numbers are gene ID annotated in Carnation DB [35)].

Iijima *et al*. Supplementary Figure S4

Supplementary Figure S5. UPLC chromatograms and MS data of flavonoid and anthocyanin extracts from stage 3 petals.

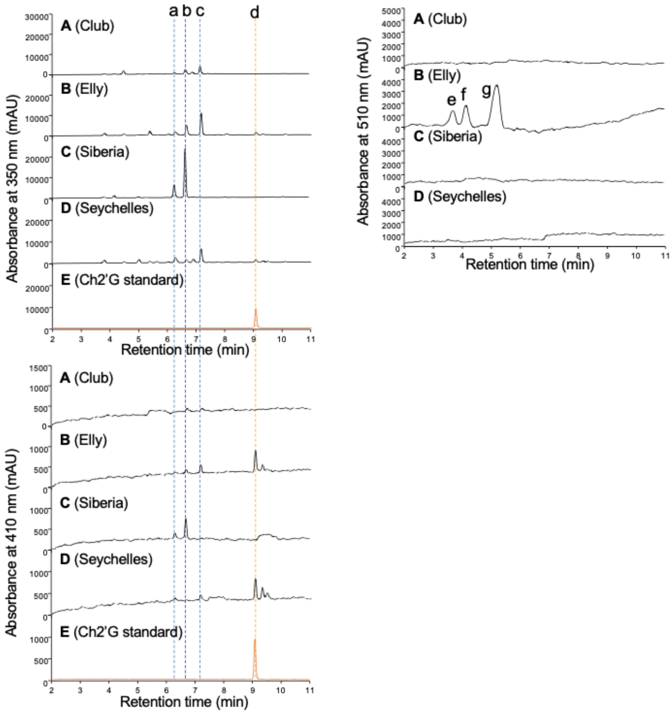

peak	identification	RT	molecular formula	molecular ions	fragments	λ_{max} (nm)
a	kaempferol 3-*O*-glucosyl-(1→2)-[rhamnosyl-(1→6)-glucoside]	6.27	$C_{33}H_{41}O_{20}$	757.2215 $(M+H)^+$	595, 433, 287	346
				755.2011 $(M-H)^-$	575	
b	kaempferol 3-*O*-sophoroside	6.66	$C_{27}H_{30}O_{16}$	611.1631 $(M+H)^+$	449, 287	350
				609.1415 $(M-H)^-$	429	
c	kaempferol 3-*O*-neohesperidoside	7.16	$C_{27}H_{30}O_{15}$	595.1683 $(M+H)^+$	449, 287	350
				593.1465 $(M-H)^-$		
d	chalcone 2'-*O*-glucoside (Ch2'G)	9.07	$C_{21}H_{22}O_{10}$	435.1309 $(M+H)^+$	273, 153	370
				433.1107 $(M-H)^-$	271, 151	
e	pelargonidin 3-malonylglucoside	3.68	$C_{21}H_{27}O_{14}$	519.1152 $(M)^+$	433	502
				517.1111 $(M-2H)^-$	431	
f	pelargonidin 3-glucoside	4.13	$C_{21}H_{21}O_{10}$	433.1160 $(M)^+$	271	502
				431.0961 $(M-2H)^-$		
g	3,5-di-(β-glucopyranosyl) pelargonidin 6''-*O*-4, 6'''-*O*-cyclic malate	5.18	$C_{31}H_{33}O_{18}$	693.1683 $(M)^+$	271	506

Supplementary Figure S5. UPLC chromatograms and MS data of flavonoid and anthocyanin extracts from stage 3 petals.

Extracts of petals of 'Club' (A), 'Elly' (B), 'Siberia' (C) and 'Seychelles' (D) were analyzed by UPLC and the elution profiles monitored by diode array detector (DAD) are shown by two dimensional panels at 350 nm, 410 nm, and 510 nm for detection of flavonols, chalcone 2′-*O*-glucoside and anthocyanins, respectively. Elution profile shown in (E) was obtained using an authentic standard for chalcone 2′-*O*-glucoside. The molecular structure of a to g were determined by MS analysis coupled with UPLC elution. a, kaempferol 3-*O*-glucosyl-(1→2)-[rhamnosyl-(1→6)-glucoside]; b, kaempferol 3-*O*-sophoroside; c, kaempferol 3-*O*-neohesperidoside; d: chalcone 2’-*O*-glucoside; e, pelargonidin 3-malonylglucoside; f, pelargonidin 3-glucoside; g, 3,5-di-(β-glucopyranosyl) pelargonidin 6″-*O*-4, 6‴-*O*-cyclic malate. The lower table shows the assignment of each peak analyzed by UPLC-MS (a to g) and their properties including retention time of UPLC elution, molecular formula, molecular ions and fragment ions of MS analysis and maximum absorption wavelengths found in a DAD equipped with UPLC.

Supplementary Figure S6. A heatmap of expression profiles of genes related to flavonoid synthesis in stage 3 petals of the four cultivars.

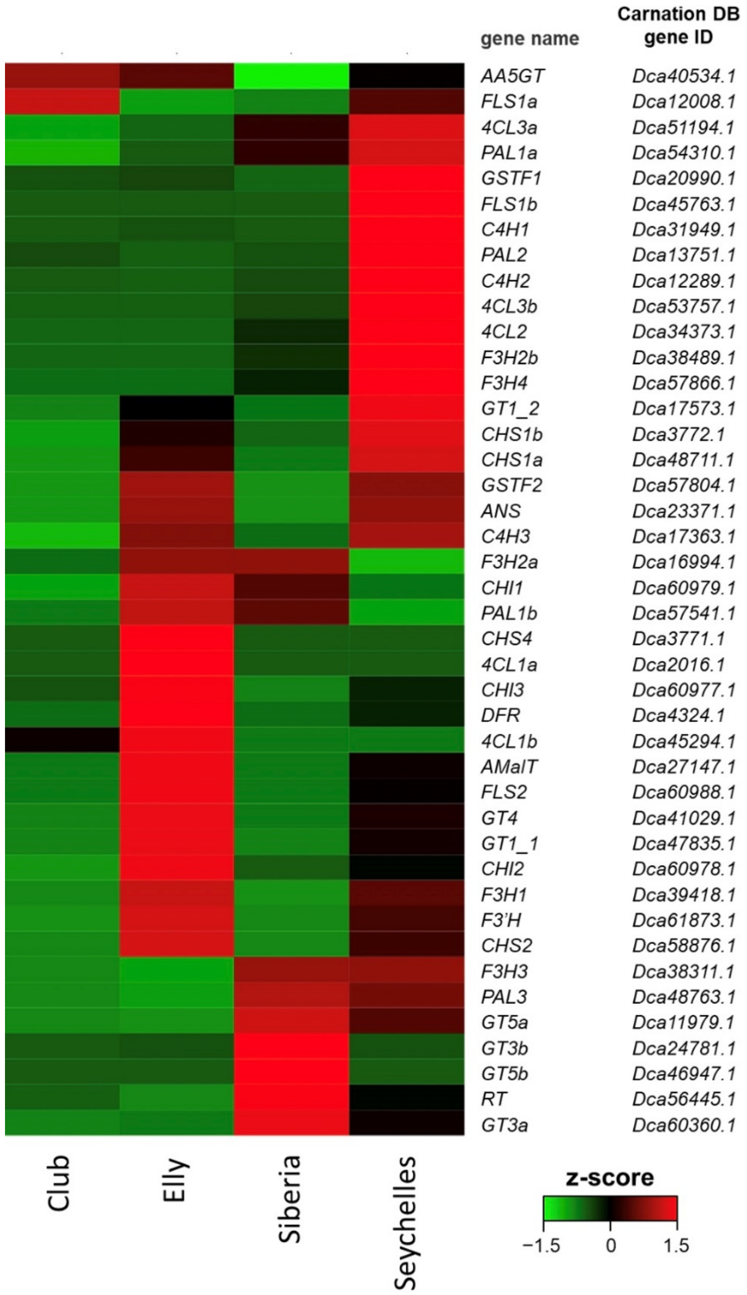

Supplementary Figure S6. A heatmap of expression profiles of genes related to flavonoid synthesis in stage 3 petals of the four cultivars.
PAL: phenylalanine ammonia lyase; C4H: cinnamate 4-hydroxylase; 4CL: 4-coumarate CoA ligase; CHS: chalcone synthase; CHI: chalcone isomerase; F3H: flavanone 3-hydroxylase; F3′H: flavonoid 3′-hydroxylase; FLS: flavonol synthase; DFR: dihydroflavonol 4-reductase; ANS: anthocyanidin synthase; GT: glucosyltransferase; AA5GT: acyl-glucose-dependent anthocyanin 5-glucosyltransferase; RT: rhamnosyltransferase; AMalT: anthocyanin malyltransferase; GST: glutathione *S*-transferase.

Supplementary Figure S7. Heatmaps of expression profiles of genes related to (A) chlorophyll synthesis, (B) chlorophyll cycle, and (C) chlorophyll degradation in stage 3 petals of the four cultivars.

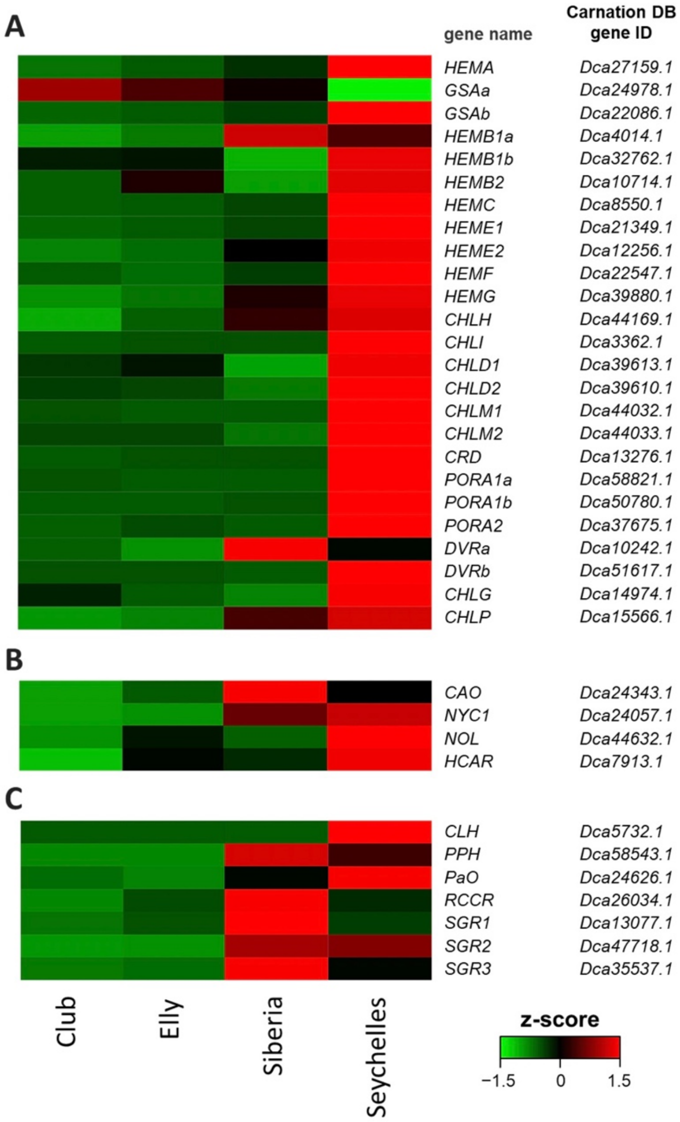

Supplementary Figure S7. Heatmaps of expression profiles of genes related to (A) chlorophyll synthesis, (B) chlorophyll cycle, and (C) chlorophyll degradation in stage 3 petals of the four cultivars.
HEMA: glutamyl-tRNA reductase; GSA: glutamate-1-semialdehyde 2,1-aminotransferase; HEMB: 5-aminolevulinate dehydrogenase; HEMC: porphobilinogen deaminase; HEME: uroporphyrinogen III decarboxylase; HEMF: coproporphyrinogen III oxidase; HEMG: protoporphyrinogen oxidase; CHLH, CHLI and CHLD: magnesium chelatase; CHLM: Mg-protoporohyrin IV methyltransferase; CRD: Mg-protoporohyrin IV monomethylester cyclase; PORA: protochlorophyllide oxidoreductase; DVR: divinyl chlorophyllide *a* 8-vinyl reductase; CHLG and CHLP: chlorophyll synthase; CAO: chlorophyllide *a* oxygenase; NYC1 and NOL: chlorophyll *b* reductase; HCAR: hydroxymethyl chlorophyll *a* reductase; CLH: chlorophyllase; PPH: pheophytinase; PaO: pheophorbide *a* oxygenase; RCCR: red chlorophyll catabolite reductase; SGR: Stay-Green.
